# Role for intravesical prostatic protrusion in lower urinary tract symptom: a fluid structural interaction analysis study

**DOI:** 10.1186/s12894-015-0081-y

**Published:** 2015-08-19

**Authors:** Junming Zheng, Jiangang Pan, Yi Qin, Jiale Huang, Yun Luo, Xin Gao, Xing Zhou

**Affiliations:** Department of Urology, The Second Affiliated Hospital of Guangzhou Medical University, 250 Changgang road, Guangzhou, 510260 China; School of Mechanical and Automotive Engineering, South China University of Technology, Guangzhou, China; Department of Urology, Third Affiliated Hospital of Sun Yat-sen University, Guangzhou, 510630 China

## Abstract

**Background:**

Numerous studies indicated that Intravesical prostatic protrusion is relevant to prognosis of LUTS, however, the confounding effect that is brought about by prostate volume, urethra anterior curvature angle and other factors makes it hard to evaluate the role of intravesical prostatic protrusion in clinical observation.

**Methods:**

We proposed a fluid structural interaction analysis approach. 3D models were constructed based on MRI images, and prostatic urethra diameters were calibrated with urodynamic data. Comparisons of urine flow dynamics were made between models with various degree of intravesical prostatic protrusion, while the intravesical pressure, anterior urethra curvature angle and diameter of prostatic urethra were same among all models to rule out their confounding effects.

**Results:**

Simulation result showed that the decrement of diameter and increment of variation in cross-sectional area for prostatic urethra were related to the degree of intravesical prostatic protrusion. Such deformation would lead to deterioration of flow efficiency and could compromise the effect of bladder outlet obstruction alleviation treatment.

**Conclusions:**

These results provided further evidence for intravesical prostatic protrusion being an independent risk factor for bladder outlet obstruction severity and demonstrated that intravesical prostatic protrusion would be a promising marker in clinical decision making.

**Electronic supplementary material:**

The online version of this article (doi:10.1186/s12894-015-0081-y) contains supplementary material, which is available to authorized users.

## Background

Intravesical prostatic protrusion (IPP) is the extent to which the prostate protrudes into the bladder, defined as distance from protruded prostate to the base of bladder, and can be measured in midline sagittal plane of the prostate [1]. Population based data indicated that 10 % of male between 40 to 79 years old had an IPP of 10 mm or greater [2]. IPP is considered as a prognostic factor for LUTS [3, 4]. And the fact that IPP can be evaluated with non-invasive trans-abdominal ultra-sound made it a promising candidate for initial assessment of LUTS patient [5]. But the mechanism underlying the relationship between IPP and bladder outlet obstruction is still unclear. One key issue the confounding effect caused by prostate volume variation and urethra curvature angle. Because they are both risk factors for LUTS severity and are closely related with IPP, it is difficult to control these confounding factors with observational study. Computational modeling on the other hand, is a promising alternate, and would shed a light on understanding the role for IPP in bladder outlet obstruction.

Hydraulic energy is the driven force in voiding process. It is lost due to resistance of urethra. Accurate reconstruction of anatomical feature for lower urinary tract is crucial for calculation of hydraulic energy loss. Computational fluid dynamic (CFD) study was proved to be advantageous in such aspect [6–8]. However, rigid wall boundary assumption in previous studies ruled out the interaction between urine flow and urethra wall movement, especially prostatic urethra wall. To overcome this limitation and investigate the role for IPP in bladder outlet obstruction, we carried out a fluid structural interaction analysis in models reconstructed from MRI data with various degree of IPP, then compared the difference in flow efficiency among these models.

## Methods

### The model and boundary conditions

A retrospective revision of the clinical data for all patients, presenting with lower urinary tract symptoms secondary to benign prostate hyperplasia (LUTS/BPH), who also completed MRI scan of pelvic region and pressure flow study before surgery, in the time period from January 2000 to December 2014 was carried out. Diagnosis of LUTS/BPH was established if criteria of the 5th International Consensus Committee on BPH [9] was met. The data from MRI scanning (Discovery MR750, GE Healthcare) are needed for model reconstruction, and the pressure flow study are needed for calibration of arbitrary determined parameters of the model. Patients with a history of neurogenic bladder, previous pelvic surgery or urinary cancer were excluded, detrusor insufficiency was also ruled out. Ten male patients were included for the study after providing informed consent. Approval for the study was granted by the ethics committee of Second Affiliated Hospital of Guangzhou Medical University.

**Fig. 1:**
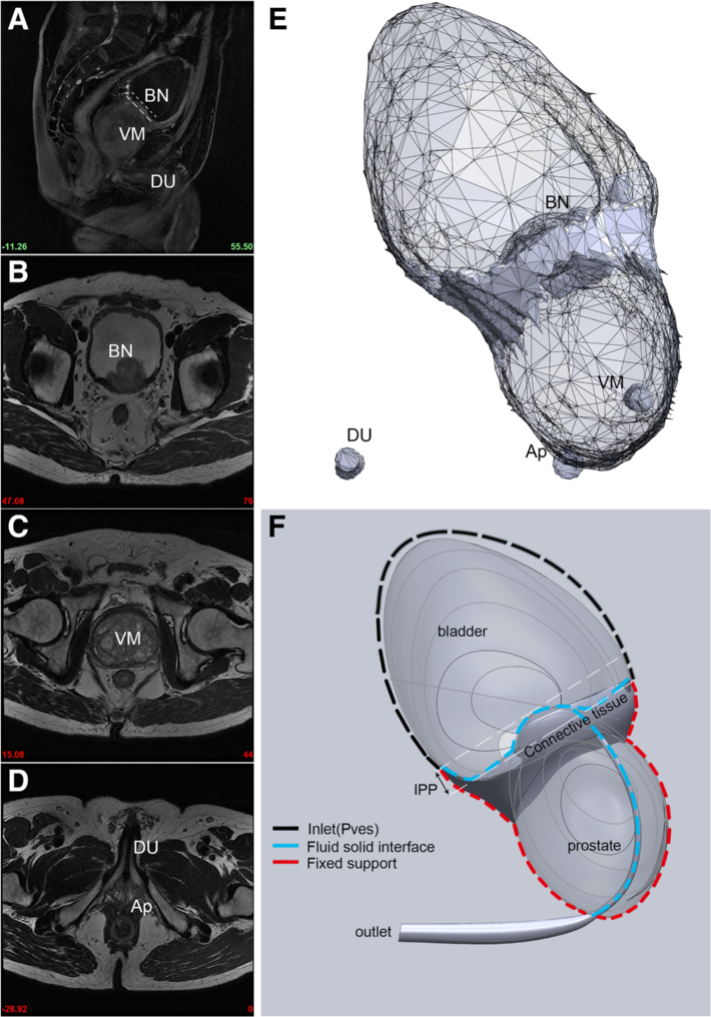
Prostate and bladder model construction MR images of lower urinary tract were collected (**a** sagittal plane, **b**-**d** axial plane, **b** bladder neck, **c**: veru montanum, **d** prostatic apex), and 3D model were reconstructed from organ contouring (**e**), and optimized (**f**). Measurement for IPP was shown in dash line in A and F. VM: veru montanum, BN: bladder neck, Ap: prostate apex, U: anterior urethra

Organ contouring for prostate, bladder and surrounding connective tissue was done in Mimics (Materialise, Leuven Belgium) by studying axial T2 MRI images of each patient. This was conducted by one senior urologist and confirmed by another radiologist. Degree of IPP was measured in mid-sagittal plane. First a line was drawn from the anterior to posterior intersections of the bladder base and prostate, then the distance between the protruded prostate to this line is defined as IPP (Fig. 1a, f), and categorized as grade I(<5 mm), grade II(5 ~ 10 mm) and grade III(>10 mm) [10]. Then three-dimensional models were constructed from contouring region of each slice (Fig. 1e), and optimized (Fig. 1f) with SolidWorks (DS Solidworks, Massachusetts, USA).

**Fig. 2:**
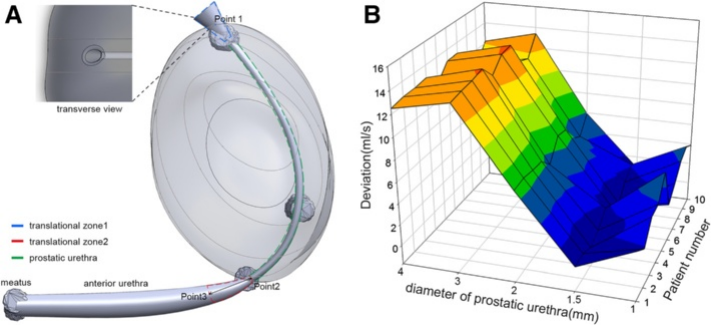
Urethra model construction and optimization (**a**) Schematic illustration of the modeling configuration for urethra. Location of Translational zone 1, translational zone 2 and prostatic urethra are marked. **b** deviation between calculated urine flow rate and measured urine flow rate

Since prostatic urethra can’t be clearly identified in MRI images [11], model was reconstructed with arbitrary parameters. The urethra model was divided into three parts. The proximal part (Fig. 2a) was a translational zone from bladder to urethra, 10 mm in length with a diameter decreasing from the width of normal bladder neck(8 mm) to prostatic urethra width [6]. The distal part (Fig. 2a) was another 10 mm long translational zone between distal prostatic and anterior urethra. The urethra in-between started at bladder neck, curved anteriorly at veru montanum and ended near at the prostatic apex [11]. These key points were marked specifically during organ contouring to define a fitting spline that would be closest to the prostatic urethra course (Fig. 1a-d). Then this part of urethra was modeled as a cylindrical structure running along the spline. The diameter for anterior urethra(d_urethra_) was set to 5 and 4 mm for meatus, corresponding to the average cross-sectional area of around 20 mm^2^ for anterior urethra [12, 13]. Diameter for prostatic urethra during voiding process was a parameter paramount for accurate simulation. The value should be between 1 mm and the diameter of anterior urethra [14]. Five candidate diameters (1, 1.5, 2, 3 and 4 mm) were proposed for initial diameter of prostatic urethra (d_prostate_). Result of Abrams-Griffiths nomogram in our pilot study showed that these 5 candidate diameter covers the pressure-flow relationship from obstructed to unobstructed scenario (Additional file 1: Figure S1). This coincided with previous result [15]. In this way, we could preserve the anatomic feature of anterior urethra curvature, and find the optimum diameter which would represent the obstruction level in prostatic urethra at the same time.

Fluid structural interaction analysis in ANSYS (ANSYS, Inc. Canonsburg, USA) was employed to study the deformation of prostate and its influence on urine flow. Boundary conditions were configured as follows (Fig. 1f): (1) Superior wall of bladder was set as inlet with preset total pressure; (2) Meatus of the urethra was set as outlet with 1 atm (101,325 Pa) static pressure; (3) Fluid-structural interface region included the bladder wall which lay over the protruded prostate, the surrounding connective tissue, the bladder neck and prostatic urethra; (4) Fixed support was added to the lateral wall of prostate and the surrounding connective tissue, representing the supportive structure around the prostate, along with fascia and pelvic supportive structures. The properties of the fluid were set as water. Pilot studies indicated that for all models in our simulation, the reynolds number, defined as Re = ρvd/η (ρ is the density of fluid, η is the dynamic viscosity of fluid, v and d are the velocity and hydraulic diameter of flow, respectively), range from 7000 to 13,000 (Additional file 2: Table S1), which were greater than 4000. So κ-ε turbulence model was used for CFD analysis. For the structural analysis, prostate was assumed to be linear elastic [16] (Poisson ratio:0.4, Young’s modulus: 21kPa). The same assumption was applied to connective tissue (Poisson ratio:0.4, Young’s modulus:15kPa).

### Simulation planning

By adopting five candidate diameter of prostatic urethra to all ten patients, 50 candidate models were reconstructed. Intravesical pressure (P_ves_) measured at maximal urine flow rate (V_m_) was used as total pressure for inlet, then flow rate (V_c_) was calculated with fluid structural analysis model. Bladder volume was set to be more than 200 ml in all models. Calculated flow rate (V_c_) and measured flow rate (V_m_) were compared to select the optimal d_prostate_.

One model from a patient with grade 2 IPP was selected by random, and marked as model 2. Another two models with different grades of IPP were created by adjusting the shape of the protruded part. These two models were marked as model 1 (grade 1 IPP model) and model 3 (grade 3 IPP model). Shape of the bladder neck, prostatic urethra diameter and anterior urethra curvature angle were same among three models (Additional file 3: Figure S2). Same boundary conditions were applied to all models. For each model, Fluid structural interaction(FSI) analysis were conducted under three different inlet pressure(7840.8, 17081.1, 13721.4 Pa), results of prostatic deformation and flow efficiency were compared among three models. Then, to simulate the obstruction alleviated scenario, initial diameter of prostatic urethra was increased to 2 and 3 mm, respectively. Then urine flow rate was recalculated under the intravesical pressure of 80cmH2O (7480.8 Pa).

## Results

The demographic data of ten patients was listed in Table 1. Pressure flow data (Additional file 4: Table S2) showed that mean maximal urine flow rate(Q_max_) was 9.5 ml (7 ~ 12 ml), detrusor pressure at Q_max_ (P_det,Qmax_) was 56.7 cmH2O (42 ~ 70 cmH2O), voided volume was 188.5 ml (153 ~ 245 ml). This result indicated that all patients can be categorized as obstructed according to Blaivas’ criteria [17]. For each patient, a model was constructed using MRI images, and urine flow rate was calculated. The deviation between calculated and measured flow rate in each candidate model were charted in a 3D mesh. Minimal deviation was acquired when d_prostate_ = 1.5 mm, and maximal deviation was found when d_prostate_ = 3 mm (Fig. 2b). Initial diameter of prostatic urethra for model 1, model 2 and model 3 were set to be 1.5 mm.

**Table 1:** Patient demographics

measure	Mean	Minimal-maximal
Sample size	10	
Age	64.1	59–70
Prostate volume(ml)	92.3	53.5–115.3
Intravesical protrusion(mm)	8.67	3.2–12.3
Maximal flow rate(ml/s)	9.5	7–12
Pves at maximal flow rate(Pa)	10,046	8722.89–11271.15

**Fig. 3:**
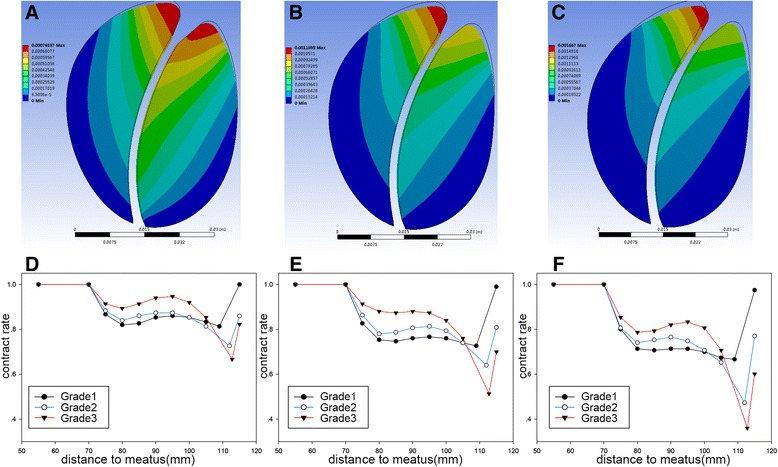
Structure analysis for prostatic deformation (**a**-**c**) sagittal cross-section view of contouring figure for deformation. Pves =140 cmH2O (13721.4 Pa), **a** grade 1 IPP, **b** grade 2 IPP, **c** grade 3 IPP. D-F: the rate of cross-sectional area constriction for 3 models, calculated by dividing the cross-sectional area of constricted urethra by original cross-sectional area at the same point. **d** Pves = 80 cmH2O (7840.8 Pa), **e** Pves = 110 cmH2O (10781.1 Pa), **f** Pves = 140 cmH2O (13721.4 Pa)

During voiding, we found that deformation of the prostate would lead to urethra constriction (Fig. 3a-c), and its magnitude increased with intravesical pressure. The constriction of urethra was most prominent near bladder neck, then loosened gradually and returned to its initial diameter at prostatic apex. Under each intravesical pressure, urethra diameter of different models was compared. Such comparison leads to an interesting discovery. In region near bladder neck, the widest urethra was found in model 1 (grade 1 IPP model), followed by model 2 and model 3(grade 3 IPP model), but this order was reversed in distal prostatic urethra. Such pattern of constriction indicated that the variation of cross sectional area for urethra was most prominent in model 3, and lowest in model 1 (Fig. 3d-f).

**Fig. 4:**
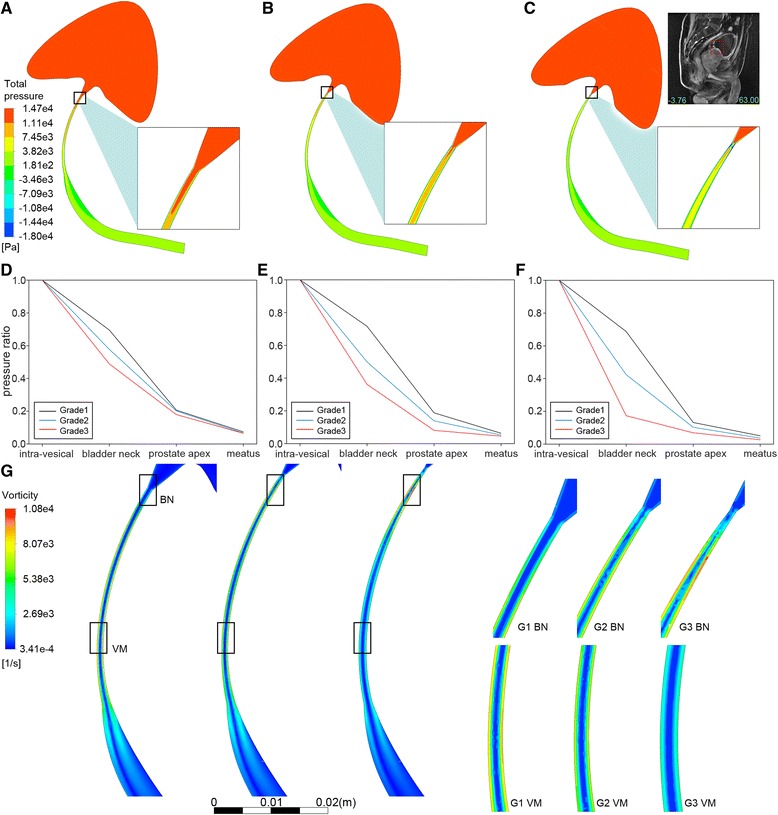
Flow efficiency analysis. **a**-**c** total pressure distribution in sagittal cross-section with zoomed view of bladder neck. Pves =140 cmH2O (13721.4 Pa), **a** grade 1 IPP, **b** grade 2 IPP, **c**: grade 3 IPP. **d**-**f** the rate of total pressure, calculated by dividing total pressure of current location with intravesical pressure, **d** Pves = 80 cmH2O (7840.8 Pa), **e** Pves = 110 cmH2O (10781.1 Pa), **f** Pves = 140 cmH2O (13721.4 Pa). **g** vorticity distribution in sagittal cross-section and zoomed view of region in bladder neck and veru montanum, Pves =140 cmH2O (13721.4 Pa). BN: bladder neck, VM: veru montanum, G1: grade 1 IPP, G2: grade 2 IPP, G3: grade 3 IPP

Total pressure, defined as $\mathrm{P}=\frac{\uprho}{2}{\overline{\mathrm{v}}}^2+\overline{\mathrm{p}}$, was a combination of static pressure and dynamic pressure, often used to evaluate flow efficiency. As urine flow runs from bladder to urethra meatus, total pressure decreased due to urethra resistance. In our simulation, such pressure loss was most prominent in model 3 (Fig. 4a-c), most of which occurred in the constricted urethra near bladder neck. In the other two models, less total pressure was loss around the bladder neck. For these two models, the majority of total pressure loss took place in distal prostatic urethra (Fig. 4d-f).

Vorticity, defined as curl of velocity [18]: $\upomega =\nabla \times \overrightarrow{\upnu}$, was used to study the pattern of flow energy dissipation. Consistent with pattern in total pressure loss, highest magnitude of vorticity for model 3 was found in bladder neck region while the highest vorticity magnitude for model 1 located in veru montanum and prostatic apex (Fig. 4g).

**Fig. 5:**
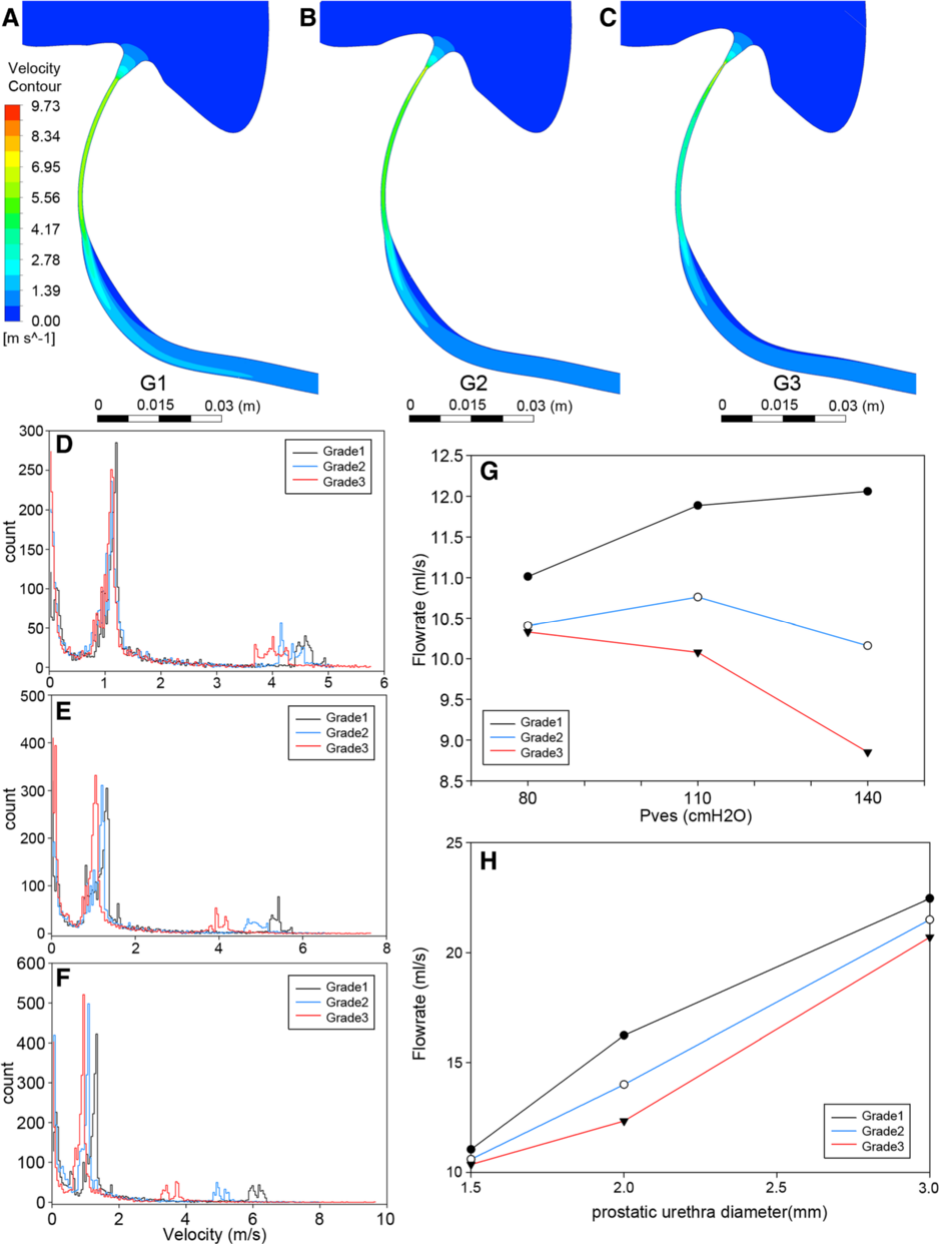
Flow velocity and flow rate analysis. **a**-**c** sagittal cross-section view of contouring figure for flow velocity, Pves =140 cmH2O, **a** grade 1 IPP, **b** grade 2 IPP, **c** grade 3 IPP. **d**-**f** histogram of flow velocity for three models. **d** Pves = 80 cmH2O (7840.8 Pa), **e** Pves = 110 cmH2O (10781.1 Pa), **f** Pves = 140 cmH2O (13721.4 Pa). **g** flow rate for all models under each particular intravesical pressure in our simulation. **h** flow rate for models with increased initial urethra diameter, calculated under the intravesical pressure of 80 cm (7840.8 Pa)

Flow velocity reached its peak as urine run through bladder neck, and then it decreased gradually (Fig. 5a-c). Histograms for flow velocity distribution in sagittal plane were compared among models (Fig. 5d-f). For each model, two peaks were found, corresponding to flow velocity in prostatic urethra and anterior urethra, respectively. Comparison among models indicated that flow velocity in majority part of model 1 was higher than the other two models.

Urine flow rate at the urethra meatus in model 1 was greater than the other two models, and increasing intravesical pressure would further widen this gap (Fig. 5g). Then, to simulate obstruction alleviated scenario, prostatic urethra diameter was increased to 2 and 3 mm for the three models. Urine flow rate was calculated in the diameter increased models under the pressure of 80 cmH2O (7840.8 Pa). We found that urine flow rate in all models increased as initial urethra diameter widen, but at different rates. The gap in flow rate between model 1 and model 3 went up to 4 ml/s when the diameter was 2 mm. Then it went down to 1 ml/s when diameter was 3 mm (Fig. 5h).

## Discussion

In patient with LUTS symptom, the confounding effect between prostate volumes, anterior urethra curvature angle, detrusor muscle contractility and intravesical prostatic protrusion (IPP) make it hard to understand IPP’s role with clinical observation. Invasive methods such as pressure flow study are generally not applicable to routine clinical practice. Computational fluid dynamic(CFD) was already proved to be an effective method in investigating flow dynamic in studies regarding airway flow [19], circulation [20] and urine transport [21]. It is an attractive alternative to elucidate the role for IPP in LUTS manifestation.

This is the first study to investigate voiding behavior of lower urinary tract with fluid structural interaction analysis (FSI) and provide a scope for better understanding of prostate deformation. Our results showed that intravesical pressure above 7840.8 Pa was enough to cause prominent deformation of the prostate in model 3, which would lead to constriction of prostatic urethra. Clinical studies showed that maximum intravesical pressure in LUTS patient is usually between 8820.9 to 14701.5 Pa [22, 23]. So it was obvious that for LUTS patient with severe intravesical prostatic protrusion, intravesical pressure during voiding would cause the prostatic deformation which would lead to severe constriction of prostatic urethra.

Structural analysis showed that the most severe constriction in bladder neck and greatest variation of cross-sectional area for urethra were found in model 3. The anatomy feature of fascia surrounding the prostate might shed a light on this. Prostate was attached anteriorly by pubo-prostatic ligaments, laterally by endopelvic-fascia, and posteriorly by Denonvilliers’ fascia [24]. As these supportive structures go superiorly, they fuse with other fascia, leaving the protruded portion of the prostate susceptible to radial component of intravesical pressure. And this may be the underlying cause for the difference in deformation between three models.

The variation for urethral cross-sectional area in model 3 was the main reason for its flow energy dissipation [25]. Such variation was most prominent near the bladder neck, which coincided with the distribution of pressure loss and vorticity magnitude. As degree of intravesical protrusion decreased, location for major pressure loss shifted towards distal part of prostatic urethra, and the amount of total pressure drop also decreased. Since factors other than intravesical prostatic protrusion were same among three models, our results indicated that intravesical prostatic protrusion could affect the flow efficiency independently.

A non-linear relationship between intravesical pressure and maximum urine flow rate (Q_max_) was found in our simulation. While flow rate in model 1 increased along with intravesical pressure, it decreased in model 3. This was clinical relevant since patient with LUTS often tend to strain to pass urine. The results in our study demonstrated that for patients with grade 3 intravesical prostatic protrusion (IPP), this could further aggravate the symptom of weak urine stream, while the increased intra-abdominal pressure predispose patients to complications including hernia and hemorrhoids [26, 27].

The simulation result for obstruction alleviated scenario also suggested that treatment outcome differs between patients with different grade of intravesical prostatic protrusion. Although a major relieve of obstruction could greatly increase the flow efficiency for all models, the raise in flow rate for model 3 was only half of that for model 1 when the relief of obstruction was relatively minor. This coincide with the finding that alpha blocker treatment is more effective in patients with mild IPP than in those with moderate or severe IPP [28].

Although our work presents some interesting finding, there are some limitations. All models were constructed and adjusted based on data acquired from Asian patients retrospectively which need confirmation in a larger population involving African and Caucasian patients. The future scope of our research is to confirm the relationship between FSI results and treatment response through a larger multicenter study.

## Conclusions

3D model of lower urinary tract was constructed from MRI images and adjusted according to urodynamic data. Fluid-structural interaction analysis was implemented. Results demonstrated that intravesical prostatic protrusion (IPP) predisposed the prostate to the deformation caused by intravesical pressure. The constriction of prostatic urethra and increased variation of cross-sectional area around bladder neck would lead to deterioration of urine flow efficiency, and compromise the effect of obstruction alleviation treatment. This study provided further evidence suggesting that IPP influence bladder outlet obstruction independently, and the flow efficiency deterioration was more resistant to obstruction alleviation treatment as the degree of IPP increased.

## Additional files

Additional file 1: Figure S1. The relationship between pressure and flow rate for five sets of prostatic urethra diameters. (JPEG 175 kb) Additional file 2: Table S1. Reynolds number of each patient was calculated for all five candidate urethra diameters. (JPEG 109 kb) Additional file 3: Figure S2. A: Cross section in sagittal plane showing the original model (model 2) and the other two models (model 1 and model 3) share the same urethral path, B: 3 models have the same initial shape for bladder neck. (PDF 30 kb) Additional file 4: Table S2. Result for pressure flow studies. (PDF 37 kb)

## Abbreviations

IPP: Intravesical prostatic protrusion
LUTS: Lower urinary tract symptom
LUTS/BPH: Lower urinary tract symptom secondary to benign prostatic hyperplasia
FSI: Fluid structural interaction analysis
CFD: Computational fluid dynamic
MRI: Magnetic Resonance Imaging
atm: The standard atmosphere
Q_max_: Maximal urine flow rate
P_det,Qmax_: Detrusor pressure at maximal urine flow rate
P_ves_: Intravesical pressure
3D: Three dimensional
