# Supplementary figures and images for: Role for intravesical prostatic protrusion in lower urinary tract symptom: a fluid structural interaction analysis study
Source: BMC Urol. 2015 Aug 19;15:86. doi: 10.1186/s12894-015-0081-y (PMC4543472; doi:10.1186/s12894-015-0081-y)

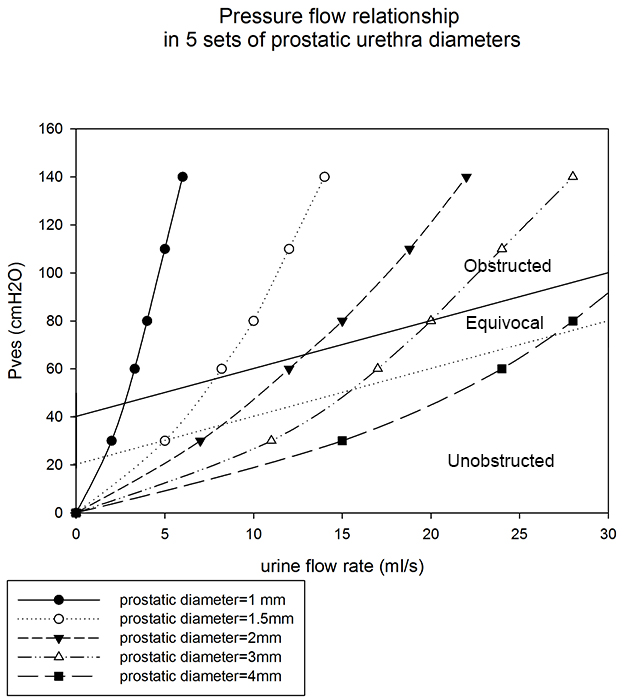

Supplement: Additional file 1: Figure S1. — The relationship between pressure and flow rate for five sets of prostatic urethra diameters. (JPEG 175 kb) [file 12894_2015_81_MOESM1_ESM.jpeg]

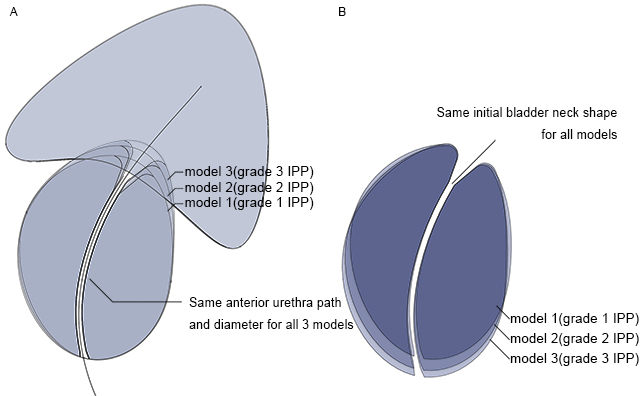

Supplement: Additional file 2: Table S1. — Reynolds number of each patient was calculated for all five candidate urethra diameters. (JPEG 109 kb) [file 12894_2015_81_MOESM2_ESM.jpeg]
