# Supplementary material for: Role for intravesical prostatic protrusion in lower urinary tract symptom: a fluid structural interaction analysis study
Source: BMC Urol. 2015 Aug 19;15:86. doi: 10.1186/s12894-015-0081-y (PMC4543472; doi:10.1186/s12894-015-0081-y)
Supplement: Additional file 3: Figure S2. — A: Cross section in sagittal plane showing the original model (model 2) and the other two models (model 1 and model 3) share the same urethral path, B: 3 models have the same initial shape for bladder neck. (PDF 30 kb) [file 12894_2015_81_MOESM3_ESM.pdf]

Table 1 in supplement data
Reynold numbers(Re= ρvd/η)

model number	hydraulic diameter(mm)	average flow velocity(m/s)	Reynold numbers
1	1	11.3	12671.23596
	1.5	6.7	11269.55056
	2	5.4	12110.5618
	3	3.4	11437.75281
	4	1.9	8522.247191
2	1	8.8	9867.865169
	1.5	5.6	9419.325843
	2	4.5	10092.13483
	3	3	10092.13483
	4	1.8	8073.707865
3	1	8.4	9419.325843
	1.5	5.6	9419.325843
	2	4.6	10316.40449
	3	3.1	10428.53933
	4	1.8	8073.707865
4	1	11.1	12446.96629
	1.5	6.6	11101.34831
	2	5.2	11662.02247
	3	3.3	11101.34831
	4	1.9	8522.247191
5	1	6.3	7064.494382
	1.5	4.4	7400.898876
	2	4.1	9195.05618
	3	2.7	9082.921348
	4	1.6	7176.629213
6	1	12.4	13904.7191
	1.5	7.1	11942.35955
	2	5.7	12783.37079
	3	3.5	11774.1573
	4	2.1	9419.325843
7	1	9.2	10316.40449
	1.5	6.2	10428.53933
	2	4.8	10764.94382
	3	3.1	10428.53933
	4	1.9	8522.247191
8	1	8.8	9867.865169
	1.5	5.6	9419.325843
	2	4.5	10092.13483
	3	3	10092.13483
	4	1.8	8073.707865
9	1	8.6	9643.595506
	1.5	5.1	8578.314607
	2	4.7	10540.67416
	3	3.1	10428.53933
	4	1.8	8073.707865
10	1	7.1	7961.573034
	1.5	5.1	8578.314607
	2	4.3	9643.595506
	3	3	10092.13483
	4	1.7	7625.168539
minimal			7064.494382
maximal			13904.7191
