# Supplementary material for: Role for intravesical prostatic protrusion in lower urinary tract symptom: a fluid structural interaction analysis study
Source: BMC Urol. 2015 Aug 19;15:86. doi: 10.1186/s12894-015-0081-y (PMC4543472; doi:10.1186/s12894-015-0081-y)
Supplement: Additional file 4: Table S2. — Result for pressure flow studies. (PDF 37 kb) [file 12894_2015_81_MOESM4_ESM.pdf]

Table 2 in supplement data
Result for pressure flow studies

pt number	max flow rate(ml)	Pves at maximal flow rate(cmH2O)	Pdet at maximal flow rate(cmH2O)	voided volume(ml)
1	11	110	54	207
2	9	105	70	182
3	9	95	45	245
4	11	115	64	214
5	7	98	56	164
6	12	104	69	177
7	10	101	53	153
8	9	113	56	168
9	9	89	58	195
10	8	95	42	180
average	9.5	102.5	56.7	188.5
minimal	7	89	42	153
maximal	12	115	70	245
